# Are pulmonary neuroepithelial bodies sensors for acute airway hypoxia implicated in the central regulation of breathing?

**DOI:** 10.1371/journal.pone.0351688

**Published:** 2026-06-23

**Authors:** Inge Brouns, Kathy Schnorbusch, Isabel Pintelon, Robrecht Lembrechts, Ian De Proost, Paul J. Kemp, Jean-Pierre Timmermans, Dirk Adriaensen

**Affiliations:** 1 Laboratory of Cell Biology and Histology, Department of Veterinary Sciences, University of Antwerp, Antwerpen, Belgium; 2 Antwerp Centre for Advanced Microscopy (ACAM), University of Antwerp, Antwerpen, Belgium; 3 School of Biosciences, Cardiff University, Cardiff, Wales, United Kingdom; Anhui University of Chinese Medicine, CHINA

## Abstract

Although their functions have long been disputed, pulmonary neuroepithelial bodies (NEBs) are now considered complex, multifunctional units implicated in vagal sensory signaling within the brain–lung axis. A widely proposed function of NEBs is that their neuroendocrine cells would be able to sense acute airway hypoxia, triggering Ca² ⁺ -dependent transmitter release and the subsequent activation of vagal afferents that transfer the hypoxic information to the central nervous system (CNS). However, physiological evidence for the latter well-documented hypothesis is so far inconclusive. Using a confocal live-cell imaging model, based on murine precision-cut lung slices (PCLSs), this study was designed to directly visualize hypoxia-induced activation of NEB cells, including associated Ca² ⁺ -mediated exocytotic events that would support CNS-directed signaling. In PCLSs from prenatal and postnatal C57BL/6 mice, including GAD67-GFP mice, we monitored changes in intracellular Ca²⁺ ([Ca²⁺]_i_), mitochondrial membrane potential, and reactive oxygen species (ROS) during acute and intermittent hypoxia, as well as after ROS scavenging. Whole-mount mouse carotid bodies served as positive controls. Carotid body glomus cells showed robust hypoxia-induced [Ca²⁺]_i_ rises, confirming assay sensitivity. In contrast, neither acute (2 or 12% O₂) nor intermittent hypoxia elicited [Ca²⁺]_i_ increases in NEBs or delayed activation of adjacent Clara-like cells at any developmental stage. NEBs remained responsive to K^+^-induced depolarization, though excitability appeared to decrease during hypoxia. Hypoxia caused rapid, reversible mitochondrial depolarization in NEBs and ciliated epithelial cells, accompanied by a modest ROS increase in all airway epithelial cells. Tempol did not uncover any [Ca²⁺]_i_ responses. Whereas control airway epithelium and carotid body expressed all NADPH oxidase subunits, the NEB microenvironment appeared to lack clear expression of several components. We conclude that mouse NEBs do not exhibit Ca² ⁺ -mediated exocytotic responses to hypoxia and that NADPH oxidase is unlikely to function as their O₂ sensor. These findings challenge a direct NEB-to-brain signaling pathway for acute hypoxia, but support local, paracrine functions related to airway oxygenation.

## Introduction

Recent advances in imaging technologies, molecular tools, and genetically modified mouse models have greatly renewed interest in (vagal) sensory pathways in lungs [1–5, for review see 6,7]. This resurgence has also extended to pulmonary neuroepithelial bodies (NEBs) [8] and their extensive intraepithelial terminals formed by myelinated vagal afferents [1,2,4,9]. Although the functional significance of pulmonary NEBs has been debated for over a century [1,2,4,10–23], it is now increasingly recognized that NEBs represent complex multifunctional units integral to the brain-lung axis [for review see 7,24–27], with potential roles in regulating physiological processes such as breathing and heart rate [25].

Pulmonary NEBs [28] are organized as intricately innervated clusters of pulmonary neuroendocrine cells (PNECs), that act as transducers of environmental stimuli. From their initial discovery [29,30], PNECs were noted to contain dense-cored vesicles (DCVs), later shown to store a wide variety of bioactive molecules, including neuropeptides (e.g., calcitonin gene-related peptide (CGRP) and gastrin-releasing peptide (GRP)), amines (e.g., serotonin (5-hydroxytryptamine; 5-HT), purines (e.g., ATP) and amino acids (e.g., γ-aminobutyric acid (GABA)) [17,22,31–34]. These DCVs, located mainly at the basal pole of PNECs, enable basal content secretion and may mediate paracrine interactions or signaling to underlying NEB-associated nerve terminals [17, for reviews see 23,26,27,35–38].

A few years ago, single-cell RNA sequencing on targets of PNEC signals [39], revealed that in mice the NEB-innervating pulmonary sensory neurons [4] express receptors for serotonin, GABA, angiotensin and glutamate [39], supporting the idea that transmitter release by PNECs can influence signaling to the central nervous system (CNS). Although not directly assessed in that study, earlier work demonstrated purinergic signaling from NEBs to the CNS [40–42]. Specifically, confocal live cell imaging (LCI) and pharmacological manipulation in an *ex vivo* precision-cut lung slice (PCLS) model revealed direct Ca^2+^-mediated ATP release from NEB cells (i.e. PNECs) in mice [40]. Following ATP-release by the NEB cells, the Clara-like cells (CLCs) that typically envelop the apical and lateral surfaces of the NEB cells, exhibit a delayed rise in intracellular calcium ([Ca^2+^]_i_), mediated by P2Y2 receptor stimulation [40,43–45]. This delayed Ca^2+^ response in CLCs serves as an indirect but visual readout of ATP release from NEB cells. Since NEBs in mice harbor vagal sensory myelinated nerve terminals expressing P2X2/3 [19] and P2Y1 [46] purinergic receptors, a clear route for ATP-mediated signaling to the CNS is provided.

The PCLS model offers the advantage to preserve the native spatial architecture of lung tissue, allowing functional analysis of ‘intact’ NEBs *in situ* [for review see 47]. Effective transduction, however, requires that NEB cells are capable of sensing external stimuli. Recent single-cell transcriptomic data revealed that NEB cells express diverse sensory gene combinations, suggesting multimodal sensing capabilities [39]. Consistent with this, previous physiological studies using an optimized murine PCLS model already proved that mechanical [48] and chemical [49] stimuli are able to elicit ATP release from NEB cells.

By far the most frequently proposed and extensively documented function of pulmonary NEBs is their ability to sense hypoxia in the airway lumen [for reviews see 22,36,37,50–53]. In this context, NEBs are thought to act as airway O_2_ sensors that signal the brain to increase respiratory drive and maintain adequate oxygenation [for review see 25]. Embedded in the airway epithelium with direct exposure to the airway lumen, PNECs are optimally positioned to monitor changes in the luminal gas composition [for review see 37,53,54].

Central to O_2_-chemosensing in NEB cells [50,55], and in the NEB-related immortalized small cell lung carcinoma (SCLC) cell line H146 [56–59], could be the acute inhibition of reactive oxygen species (ROS)-sensitive K^+^ channels by the lack of available oxygen [37,60]. Several studies have reported the functional expression of subunits of the NADPH oxidase complex (NOX2), a putative O_2_ sensor, in the plasma membrane of PNECs [50,61–63]. Under normoxic conditions, NOX2 activity generates ROS; during hypoxia, reduced ROS production is hypothesized to inhibit outward K^+^ currents through ROS-sensitive background K^+^ channels [55,64–66]. This decreasing K^+^ current would then depolarize the cell membrane, open voltage-gated Ca^2+^ channels [67], promote Ca^2+^-influx, and trigger exocytotic release of neurotransmitters, that in turn would activate closely apposed vagal afferents, resulting in transmission of hypoxic signals to the CNS [13,33,68].

For the detailed investigation of this proposed O_2_-sensing mechanism in PNECs, several *in vitro* and *ex vivo* models have been developed, including organ cultures [69], primary PNECs isolation [70–72] and SCLC cell lines [56]. Precision-cut vibratome slices of freshly dissected rabbit [33,55,73] and mouse [62] lungs have been used in patch-clamp experiments.

The aim of the present study was to directly visualize and record hypoxia-induced activation of NEB cells and the associated Ca^2+^-mediated exocytotic events as evidence for CNS-directed signaling, using our LCI model [40,48,49]. Lung slices were exposed to acute and intermittent hypoxia, or to ROS scavenging, while monitoring changes in [Ca^2+^], mitochondrial membrane potential and ROS, using fluorescent indicators. Experiments were performed primarily on postnatal wild-type (wt) mice and, in part, on prenatal animals, since NEB hypoxia sensitivity has been suggested to play a key role during the transition from fetal to neonatal life [15,22,36,74–76]. To exclude possible interference of the applied vital NEB dye with Ca^2+^-mediated hypoxia-signaling, we also conducted experiments in mice that harbor intrinsically GFP-fluorescent NEBs [43]. As carotid bodies exhibit well-established Ca^2+^-mediated neurotransmitter release under acute hypoxia, whole-mount mouse carotid bodies were used as a positive control for the LCI set-up. Functional data were supplemented with gene expression analyses of the NADPH oxidase subunits potentially involved in O_2_ sensing.

## Materials and methods

### Animals

Lung tissue was obtained from wild-type (wt) *C57-Black/6* mice (wt Bl6; Janvier, Bio Services, Uden, Netherlands), prenatally (gestational day (GD) 17–20; n = 12), immediately postnatally (postnatal day (PD) 0–1; n = 5), 2- to 3-week-old PD (14–20); n = 31), 6- tot 8-week old (adult; n = 3), and from B6.Cg-Tg(GAD1-EGFP)3Gfng/J mice (JAX 00763; The Jackson Laboratory, Charles River, L’Arbresle, France) at PD14–20 (n = 13). The latter is a Bl6-based GAD67-GFP knock in mouse strain, further on referred to as ‘GAD67-GFP mice’ [43]. Carotid bodies were obtained from 2- to 3-week-old wt Bl6 mice (PD14–20; n = 10). All young animals were housed with their mothers in acrylic cages in an acclimatized room (12/12h light/dark cycle; 22 ± 3°C) and were provided with water and food *ad libitum*. National and European principles of laboratory animal care were followed, and experiments were approved by the animal ethics committee of the University of Antwerp.

### Live cell imaging

#### Drugs and solutions.

A standard physiological solution was used throughout the various live cell imaging (LCI) experiments, containing (in mM): NaCl, 105; KCl, 5; CaCl_2_.2H_2_O, 1.2; MgSO_4_7H_2_O, 1; D-glucose, 11; NaHCO_3_, 25; pH 7.4 adjusted with HCl. The osmolarity of all solutions was maintained between 285 and 300 mOsmol. Solutions containing a high extracellular potassium concentration ([K^+^]_o_) were prepared by equimolar substitution of KCl for NaCl. Chemicals and drugs were purchased from Sigma-Aldrich (Bornem, Belgium), unless indicated otherwise. All stimuli were applied to lung slices that were submerged in a tissue bath (2 ml) mounted on the microscope stage, perfused by a gravity-fed system (flow rate of >5ml/min) with triggered valves that allowed the fast and reproducible exchange of solutions. All tubing was gas impermeant (tygon tubing, BDH, Atherstone, UK). Where appropriate, the pO_2_ of the physiological solution was equilibrated by bubbling with a normoxic (21% O_2_, 5% CO_2_, 74% N_2_) or hypoxic (0% O_2_, 5% CO_2_, 95% N_2_ or 10% O_2_, 5% CO_2_, 85% N_2_) gas mixture for at least 15 min prior to perfusion of the slices. The percentage O_2_ in the tissue bath solution was measured with an OXEL-1 oxygen electrode attached to an ISO-2 dissolved oxygen meter (World Precision Instruments LTD, Hertfordshire, England); percentage O_2_ ranged from ± 21% in normoxic to 1−2% or 11−12% in hypoxic solutions, further on referred to as severe and moderate hypoxia respectively. To mimic the predicted drop in ROS production during hypoxia, a normoxic physiological solution containing the ROS-scavenger 4-Hydroxy-2,2,6,6-tetramethyl piperidine 1-oxyl (Tempol) was used.

#### Preparation of lung slices.

All animals were killed by intraperitoneal injection of an overdose of sodium pentobarbital (Nembutal 200 mg/kg, CEVA Santé Animale, Brussels, Belgium) and vibratome slices were cut from separate lung lobes as previously published [43,45,77,78]. In short, lung tissue was stabilized by instillation of a 2% agarose solution (low-melt agarose; A4018, Sigma) via a tracheal cannula. After inflation, lungs were dissected and transferred to an ice-cold physiological solution to enable complete gelling of the agarose. Lung slices (100−150 µm thick) were cut using a vibratome (HM650V; Microm International, Walldorf, Germany) with cooled tissue bath (4°C). All precision-cut lung slices (PCLSs) were incubated in Dulbecco’s modified Eagle’s medium/F12 (DMEM-F-12; Gibco, Invitrogen, Thermo Fisher Scientific, Ghent, Belgium) and were used within 12 h of sacrificing the animal.

#### Visualization of NEBs in *ex vivo* lung slices.

Live staining of NEBs of wt Bl6 mice was performed as previously published [45,78]. Lung slices were incubated for 4 min with the vital dye in 4-(4-diethylaminostyryl)-N-methylpyridinium iodide (4-Di-2-ASP; 4 μM; D-289; Molecular Probes, Invitrogen, Thermo Fisher Scientific) in DMEM-F-12 at 37°C, rinsed and subsequently kept in DMEM-F-12 until studied.

#### Preparation of whole mount live carotid bodies.

After euthanasia, carotid bifurcations were isolated bilaterally and transferred to ice-cold physiological solution until the end of the dissection procedure. Carotid bodies were then incubated in DMEM-F-12 at 37°C, rinsed and subsequently kept in DMEM-F-12 in an incubator (37°C, 5% CO_2_) until further manipulation. All carotid bodies were used within 12 h of sacrificing the animal.

#### Loading procedure of different fluorescent (functional) indicators.

Loading with 4-Di-2-ASP was performed as previously published ([45,78] *see* ‘Visualization of NEBs in *ex vivo* lung slices’). For imaging [Ca^2+^]_i_, 4-Di-2-ASP-stained prenatal, postnatal and adult lung slices, and carotid bodies of wt Bl6 mice, were incubated in physiological solution containing the Ca^2+^ indicator Fluo-4 AM (10µM; Molecular Probes), for 1 h at room temperature (RT). GAD67-GFP mouse lung slices were incubated in physiological solution containing the Ca^2+^ indicator GFP-certified FluoForte (FluoForte; 10µM; ENZ-52016-5C50, Enzo Life Sciences, Zandhoven, Belgium), for 1h at RT. To perform ROS imaging, 4-Di-2-ASP-stained postnatal lung slices were incubated in physiological solution containing the ROS indicator 5-(and-6)-chloromethyl-2’,7’-dichlorodihydrofluorescein diacetate, acetyl ester (CM-H_2_DCFDA (H_2_DCF); 10µM; Molecular Probes), for 45 min at RT.

### Laser microdissection and RT-PCR

Laser microdissection (LMD) and RT-PCR of mouse lungs was performed as previously published [79]. In short, lung tissue was obtained from PD14 GAD67-GFP mice (n = 4). Lungs were dissected, immediately snap-frozen in liquid nitrogen and stored at −80°C. 20 µm thick cryosections were thaw-mounted on polyethylene terephthalate (PET) Frameslides (Leica, Wetzlar, Germany), immediately refrozen and subsequently dehydrated in a series of ethanol. Immediately afterwards, samples of NEB microenvironment (ME; i.e. PNECs, CLCs an intrinsic nerve terminals) and control airway epithelium (CAE) were excised from the slides by LMD (Leica LMD7000 system) and collected in the cap of a 0.2 ml Eppendorf tube filled with RLT Plus lysis buffer (Qiagen, Hilden, Germany). Carotid bodies were dissected from PD14–20 wt Bl6 mice (n = 5), collected in RLT Plus lysis buffer and processed together with the LMD samples as a positive control.

Total RNA of the LMD and carotid body samples was isolated using the RNeasy Plus Micro kit (Qiagen). Concentration and integrity of the RNA samples was evaluated by a 2100 Bio-analyzer (Agilent Technologies Inc., Santa Clara, CA, USA) using the Agilent RNA 6000 pico kit, typically yielding mostly intact RNA (RIN value > 6) and a concentration about 2 ng/µl (LMD samples) and 40 ng/µl (carotid bodies). cDNA was prepared using the SuperScriptIII First-Strand Synthesis SuperMix (Invitrogen) on a MJ Mini Cycler (Biorad). The PCR reaction mixture for each sample (20 µl) contained 5 µl of 1:5 diluted cDNA, 0,5 µM Forward Primer (FP) and Reverse Primer (RP) and 10 µl LC480 Probes Master Mix (containing FastStart Taq DNA polymerase). RT-PCR experiments were performed using LC480 white 96 Multiwell Plates in a LightCycler 480 (Roche Applied Science, Diegem, Belgium). Reactions were carried out as follows: after an initial denaturation-activation step at 95°C for 10 min, amplification consisted of 40 cycles of denaturation at 95°C for 10 s, annealing at 60°C for 15 s, and elongation at 72°C for 1 s, ending with a final cooling step at 40°C for 10 s. All primers were designed to be intron-spanning and to obtain an amplicon length around 150 bp (Table 1). A BLAST analysis was performed to confirm the specificity of the primers. All samples were run in triplicate and no-template controls (blanco) were included in all runs. Glyceraldehyde-3-phosphate dehydrogenase (GAP DH) was used as an internal control for all cDNA samples. Amplification products were separated on a 2% agarose gel and visualized under UV illumination.

**Table 1:** List of primers (FP = forward primer, RP = reverse primer) and probes used for RT-PCR for NADPH oxidase.

Gene	Primer/Probe	Sequence
gp91^phox^	gp91^phox^ FP	ACATCCTCTACCAAAACCATTCG
	gp91^phox^ RP	TTATGCTCTTCCAAACTCTCCG
p22^phox^	p22^phox^ FP	GCCCTCCACTTCCTGTTG
	p22^phox^ RP	TCCTTGGGTTTAGGCTCAATG
p47^phox^	p47^phox^ FP	TCATCCTTCAGACCTATCGGG
	p47^phox^ RP	ACCTCGCTTTGTCTTCATCTG
p67^phox^	p67^phox^ FP	AGTTGAGCTTCGGATTCACC
	p67^phox^ RP	CTTCAGTTCCTTGGGCTCTT

### Microscopic data acquisition

High resolution images and LCI results were obtained using an inverted microscope (Zeiss Axiovert 200; Carl Zeiss, Jena) attached to a microlens-enhanced dual spinning disk confocal system (Ultra*VIEW* ERS; PerkinElmer, Zaventem, Belgium), equipped with an argon-krypton laser (488 and 568 laser lines) or a Nikon Eclipse Ti-E inverted microscope attached to an Ultra*VIEW* VoX system (PerkinElmer), equipped with 488 and 561nm diode lasers, for excitation of green and red fluorophores. For physiological LCI experiments, lung slices were transferred to a perfusion chamber on the microscope stage and were restrained with a golden ring spanned with a sheet of nylon mesh. To avoid phototoxicity and photobleaching, laser illumination was kept to a minimum. Time-lapse images of changes in Fluo-4 (2 images/sec) and H_2_DCF (3 images/min) were captured using the 488 nm laser line for excitation, while the emitted fluorescence was selected by an emission filter for green light (band pass 500–555). Changes in FluoForte and 4-Di-2-ASP (2 images/sec) were recorded using the 561/568 nm laser line for excitation and an emission filter for red light (band pass 580–650).

### Data analysis

Images and time-lapse recordings were acquired and processed using Volocity 6.0.1 software (Improvision, PerkinElmer). For analysis of the time-lapse recordings, individual images were studied as grey value datasets. Regions of interest (ROIs) were drawn manually around identified cells or cell groups of interest. For every ROI, the fluorescence intensity, expressed as arbitrary units (A.U.), was plotted against time. To facilitate interpretation of the results, grey values were set to zero for the basal level of fluorescence that was present at the start of imaging in each ROI. 4-Di-ASP, Fluo-4, FluoForte and H_2_DCF are non-ratiometric dyes, and all changes in fluorescence values should be seen as qualitative changes in mitochondrial membrane potential, [Ca^2+^]_i_ and intracellular ROS levels, respectively.

The included traces show representative examples of the average changes in fluorescence intensity of all cells in a single ROI. Each condition has been tested for multiple ROIs in different slides of several mice.

## Results

The non-ratiometric probes used in the different experiments do not allow quantification nor comparison of the visualized parameter between different experiments. However, their qualitative use consistently yielded similar results under severe (2%) and moderate (12%) hypoxic conditions. To avoid redundancy and reduce the number of figures, we opted to present a single and representative graph/image for most of the experiments.

### Influence of acute hypoxia on the mouse carotid body: a proof of concept

To verify that our LCI set-up was able to visualize hypoxia-induced changes in [Ca^2+^]_i_ in mouse carotid body glomus cells, carotid bifurcation whole mounts (PD14−20; n = 5) were loaded with the Ca^2+^ indicator Fluo-4. To confirm proper dye loading and responsiveness, a positive control stimulus was applied. Elevating [K^+^]_o_ in the perfusion bath to 50mM for 5 s – an established positive control stimulus for pulmonary NEB cells in our LCI set-up – induced a transient increase in Fluo-4 fluorescence in glomus cell clusters of the mouse carotid body. Not all clusters exhibited equally distinct responses. Following stimulation, Fluo-4 fluorescence in responding glomus cells returned to baseline levels within minutes.

**Fig 1:**
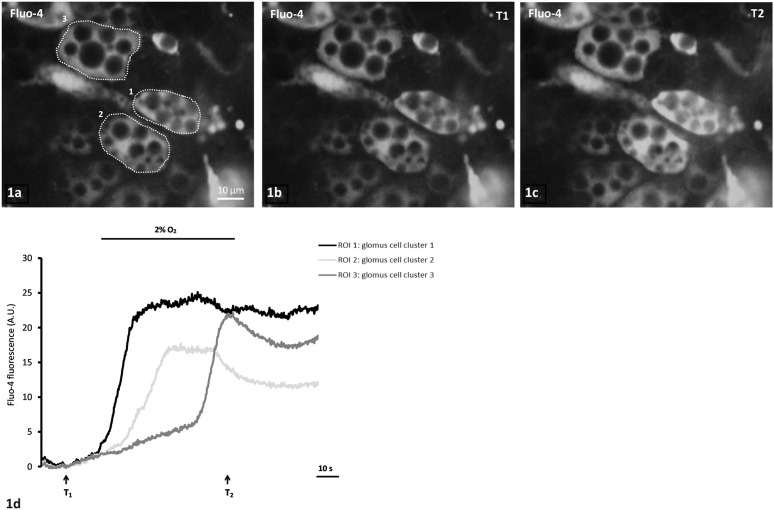
Hypoxic challenging: calcium imaging in carotid body glomus cells. Representative recording of Fluo-4 fluorescence changes in glomus cell clusters of a Fluo-4 loaded murine carotid body whole mount preparation (PD14) before and during hypoxic challenge (60 s; 2% O_2_). **a.** Initial Fluo-4 image showing loaded glomus cell clusters. **b-c.** Time-lapse images corresponding to time points T_1_ and T_2_ indicated in **(d).** Time course of Fluo-4 fluorescence intensity for three regions of interest (ROIs 1-3) corresponding to the clusters marked in **(a)**. Several glomus cell clusters respond to decreased O_2_ availability with increased Fluo-4 fluorescence.

The well-documented concept of a hypoxia-induced [Ca^2+^]_i_ rise in carotid body glomus cells was evaluated by replacing the normoxic physiological solution with a severely (2% O_2_) hypoxic solution for up to 60s. This elicited a marked increase in Fluo-4 fluorescence in glomus cell clusters, reflecting a rise in [Ca^2+^]_i_ (Fig 1a-d). The intensity and onset of this rise slightly varied between clusters, but could always be clearly detected.

To assess whether the vital NEB marker 4-Di-2-ASP – used in our established NEB LCI model – affects the O_2_-sensing mechanism, glomus cells were loaded with 4-Di-2-ASP prior to hypoxic challenge. No differences were observed in the magnitude or kinetics of the Fluo-4 fluorescence response to severe hypoxia.

### Influence of acute hypoxia on the NEB microenvironment

#### Ca^2+^-mediated responses in postnatal NEB cells.

**Fig 2:**
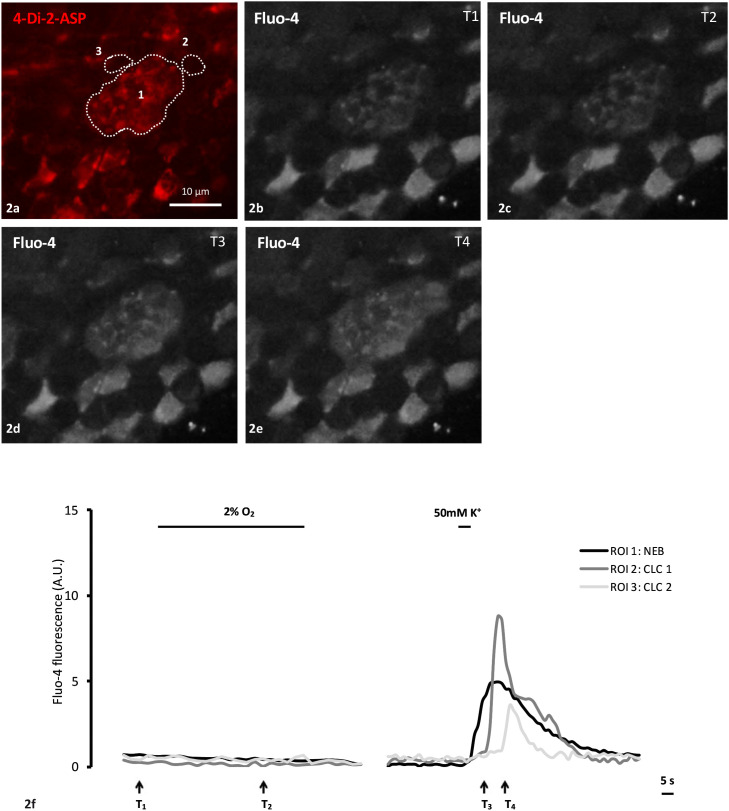
Hypoxic challenge: calcium imaging in NEBs. Representative changes in Fluo-4 fluorescence in the NEB microenvironment (NEB ME) in murine lung slices (wt Bl6; PD18) during exposure to severe hypoxia (2% O_2_; 60 s), followed by depolarization with 50mM [K^+^]_o_ (5 s). **a.** 4-Di-2-ASP staining identifies a NEB (ROI 1) distinguishable from surrounding non-fluorescent Clara-like cells (CLCs; ROI 2 and 3). **b-e.** Time-lapse images at time points indicated in **(f)**. **f.** Time course of Fluo-4 fluorescence intensity. NEB cells and CLCs do not respond to hypoxia. NEB cells (ROI 1) exhibit a robust [Ca²⁺]ᵢ elevation upon K⁺ stimulation, and CLCs the typical [40] slightly delayed [Ca²⁺]ᵢ rise (ROIs 2 and 3).

**Fig 3:**
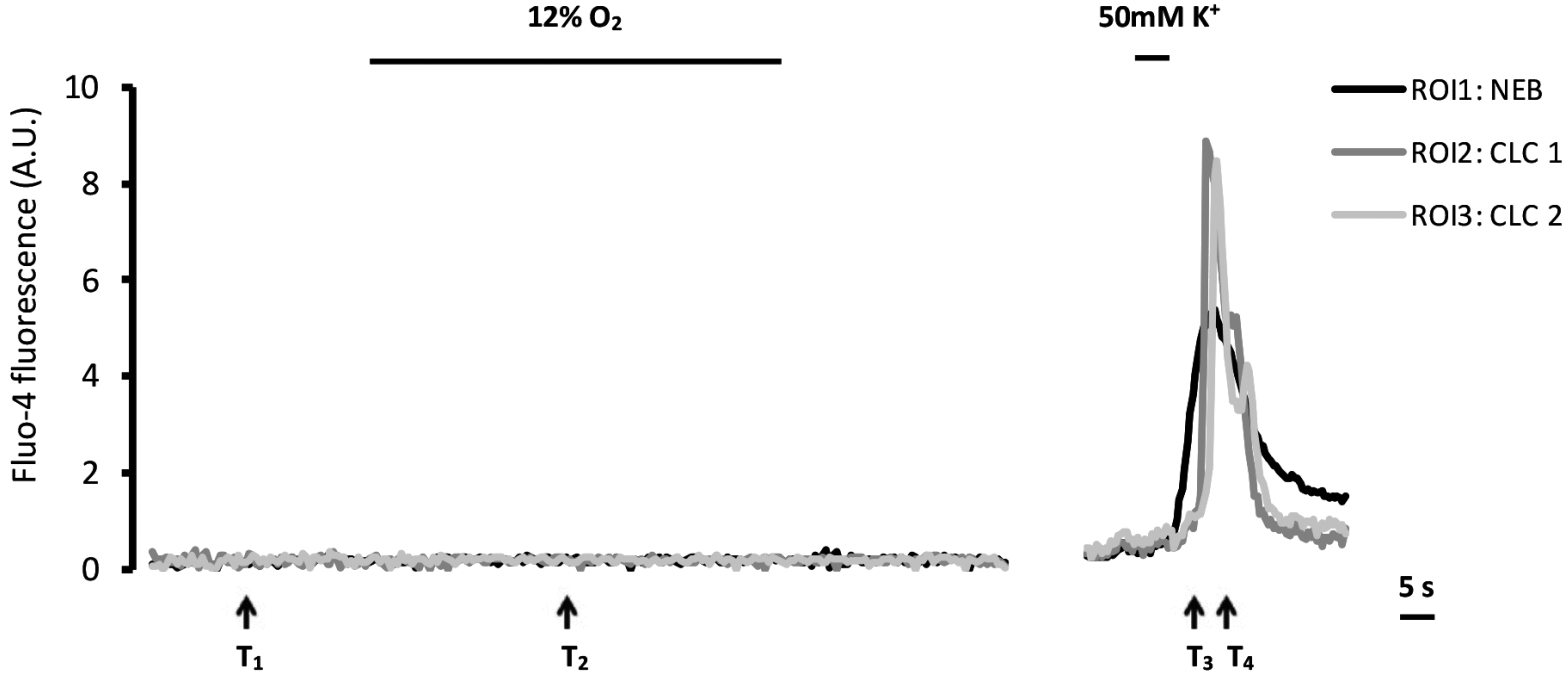
Hypoxic challenge: calcium imaging in NEBs. Representative recording of changes in Fluo-4 fluorescence measured in NEB cells and surrounding CLCs in a 4-Di-2-ASP stained, Fluo-4 loaded murine lung slices (wt Bl6; PD18) challenged with a moderate hypoxic solution (12% O_2_; 60 s) followed by high [K^+^]_o_ (50mM; 5 s). Graph plotting the time course of Fluo-4 fluorescence intensity, ROI 1-3 in the graph correspond to a NEB (ROI 1) and CLCs (ROI 2 and 3). Neither NEB cells nor CLCs respond to hypoxia, while the K^+^ stimulus elicits a characteristic rise in Fluo-4 fluorecence (i.e., a Ca^2+^ response), confirming viability and ATP release by the NEB cells resulting in a slightly delayed rise in Fluo-4 fluorescence, hence a purinergic activation of CLCs.

To examine whether acute hypoxia triggers Ca^2+^-mediated responses in NEB cells, lung slices from postnatal wt mice [PD14–20 (n = 16), PD0–1 (n = 5); adult (n = 3)] were exposed to severely (2% O_2_; Fig 2a-f) or moderately (12% O_2_; Fig 3) hypoxic solutions. Regardless of the exposure duration (between 60 and 300 s), acute hypoxia did not evoke any increase in Fluo-4 fluorescence, so no rise in [Ca^2+^]_i_ in NEB cells. In contrast, subsequent short-term depolarization with high [K^+^]_o_ (50mM; 5 s), a validated positive control [40], induced a clear increase in Fluo-4 fluorescence in all NEB cells, confirming that NEB cells were viable and properly loaded with Fluo-4. As studied and published in detail before [40], the subsequent delayed increase in Fluo-4 fluorescence of surrounding CLCs (Figs 2 and 3) reflects the Ca^2+^-mediated quantal ATP release from NEB cells followed by the purinergic activation of the CLCs. The latter proves that NEB cells release ATP that can be picked up by vagal afferents within the NEB ME and transduce the sensory information toward the central nervous system [40,48,49].

**Fig 4:**
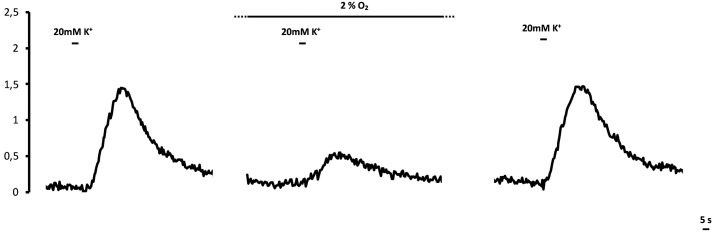
Excitability of NEBs during hypoxic challenge. Representative recording of changes in Fluo-4 fluorescence in a NEB before (normoxic conditions), during (severely hypoxic solution; 2% O_2_; 300 s) and following (normoxic conditions) perfusion of murine lung slices (wt Bl6; PD14). NEBs appear to have a much less pronounced response to high [K^+^]_o_ (20mM; 5 s) during hypoxic exposure, but this response appears to be restored upon returning to normoxic conditions.

During hypoxic exposure, depolarization with high [K^+^]_o_ (5 s) evoked a clearly smaller [Ca^2+^]_i_ response in NEB cells than under normoxia, which was restored upon reoxygenation (Fig 4).

**Fig 5:**
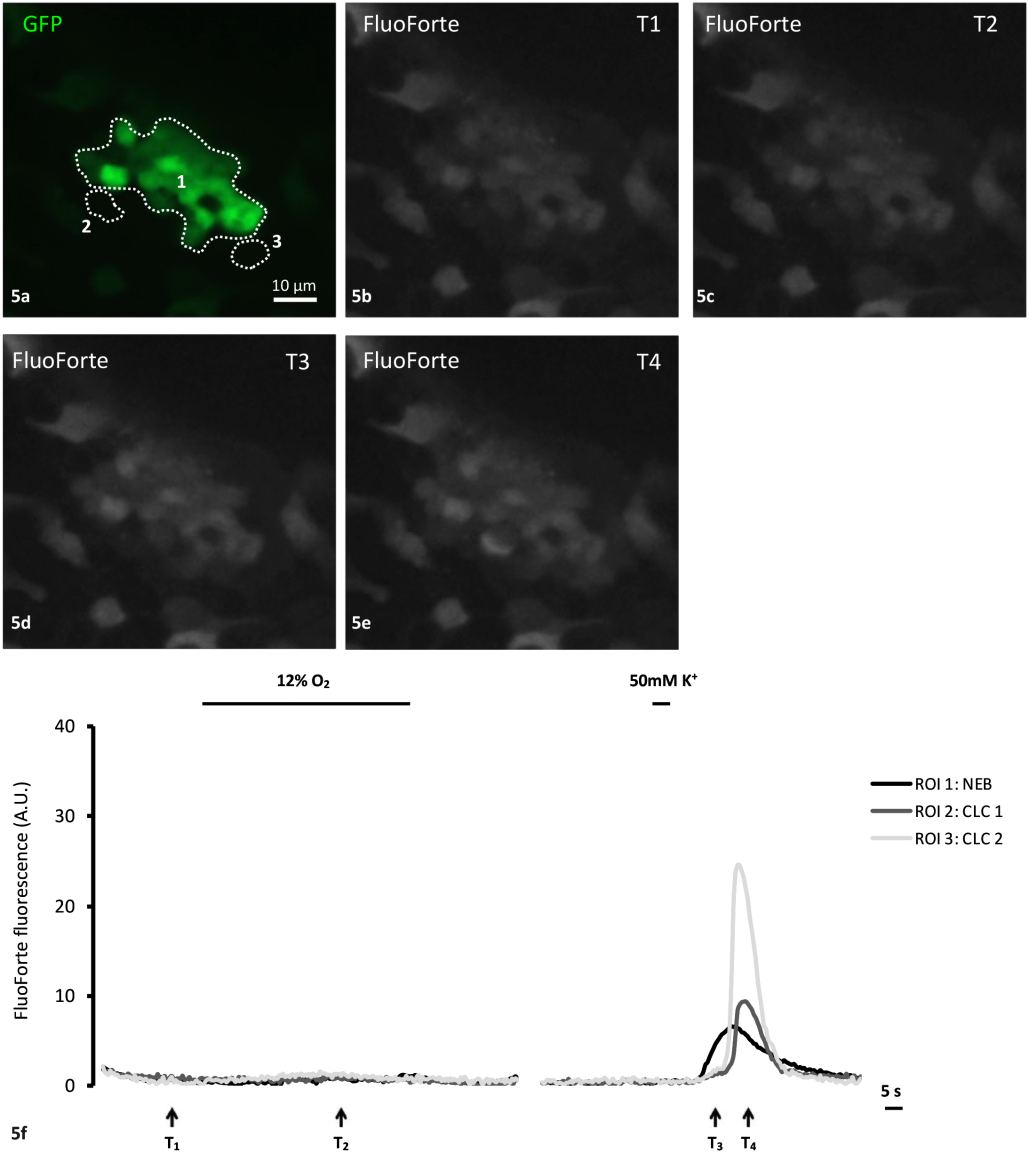
Hypoxic challenge: calcium imaging in NEB cells without 4-Di-2-ASP. Representative recording of FluoForte fluorescence changes measured in GFP-fluorescent NEB cells (ROI 1) and surrounding CLCs (ROI 2 and 3) during a 60 s challenge with 12% O_2_ and subsequent short-term (5 s) control activation with 50mM [K^+^]_o_ in a FluoForte loaded lung slice of a GAD67-GFP mouse (PD14). **a.** *Green* channel showing an image of the GFP-expressing NEB, taken prior to the experiment. **b-e.** Time-lapse images of FluoForte fluorescence in the NEB ME at time points indicated in **(f)**. **f.** Time course of changes in FluoForte fluorescence intensity. Hypoxic exposure did not lead to an increase in FluoForte fluorescence intensity in NEB cells or CLCs. The positive control shows that the NEB cells were viable, properly loaded with FluoForte, and able to induce a delayed activation of CLCs.

GAD67-GFP mouse (n = 6; PD14−20) lung slices, which harbor GFP-fluorescent NEB cells, do not require 4-Di-2-ASP staining for identification of NEBs [43]. Also in these preparations, hypoxic challenge did not lead to an increase – and thus corresponding intracellular calcium rise – in fluorescence of the red-fluorescent calcium indicator FluoForte. Subsequent short-term application of high [K^+^]_o_ (50mM; 5 s) evoked a clear and reversible rise in FluoForte fluorescence, confirming viability (Fig 5).

#### Ca^2+^-mediated responses in prenatal NEB cells.

**Fig 6:**
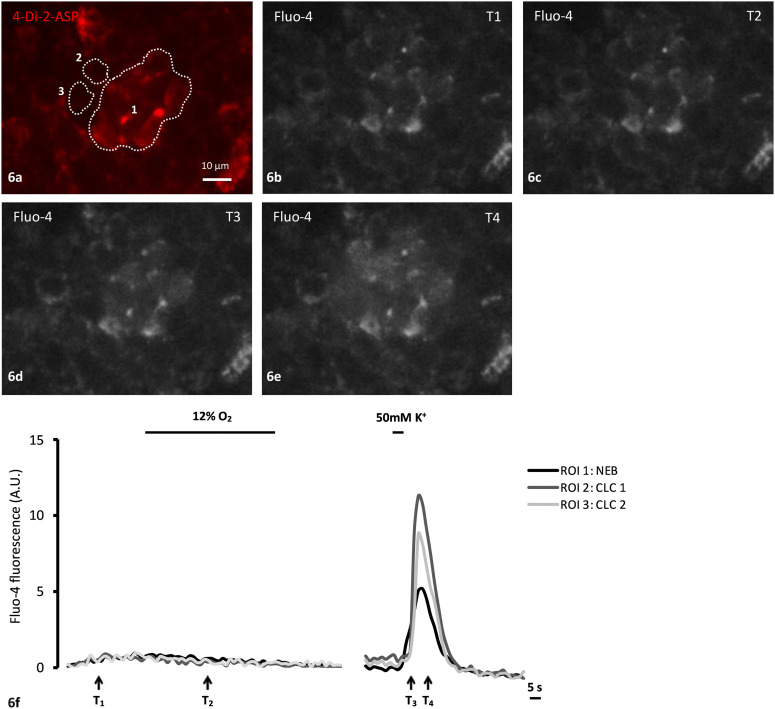
Hypoxic challenge: calcium imaging in prenatal NEBs. Representative recording of changes in Fluo-4 fluorescence measured in fetal NEB cells and surrounding CLCs in a 4-Di-2-ASP stained, Fluo-4 loaded fetal murine lung slice (wt Bl6: GD19), sequentially challenged with a moderately hypoxic solution (12% O_2_; 60 s) and high [K^+^]_o_ (50mM; 5 s). **a.** Image of 4-Di-2-ASP staining recorded at the beginning of the experiment, showing a fetal NEB (ROI 1) that can be differentiated from the surrounding non-fluorescent CLCs (ROI 2 and 3). **b-e.** Time-lapse images of Fluo-4 fluorescence in the NEB ME at time points, indicated in **(f)**. **f.** Time course of Fluo-4 fluorescence intensity. Fetal NEB cells and CLCs do not respond to moderate hypoxia, but high [K^+^]_o_ stimulation shows that the fetal NEB cells were viable, properly loaded with Fluo-4 and able to induce a delayed activation of the CLCs.

To assess whether fetal mice (GD 17−20; n = 9) NEB cells exhibit Ca^2+^-mediated responses to hypoxia, lung slices were perfused for up to 3 min with moderately or severely hypoxic solutions (Fig 6). No increase in Fluo-4 fluorescence was detected, indicating the absence of hypoxia-induced Ca^2+^ entry or neurotransmitter release. Short-term depolarization with high [K^+^]_o_ (50mM; 5 s), however, produced the Ca^2+^ response in all fetal NEB cells and the subsequent delayed activation of adjacent CLCs, confirming proper loading and functional integrity [45].

#### Mitochondrial membrane potential responses in NEB cells.

We used changes in 4-Di-2-ASP fluorescence intensity to assess changes in the mitochondrial membrane potential. In healthy live cells, mitochondria maintain a significant negative electrochemical potential across their inner membrane. This strong negative charge actively drives the accumulation of the positively charged (cationic) molecule 4-Di-2-ASP within the mitochondrial matrix. The high concentration of dye in mitochondria then partly quenches fluorescence, while mitochondrial membrane depolarization causes the release of dye in the cytoplasm and hence an over-all fluorescence rise in the visualized cells.

**Fig 7:**
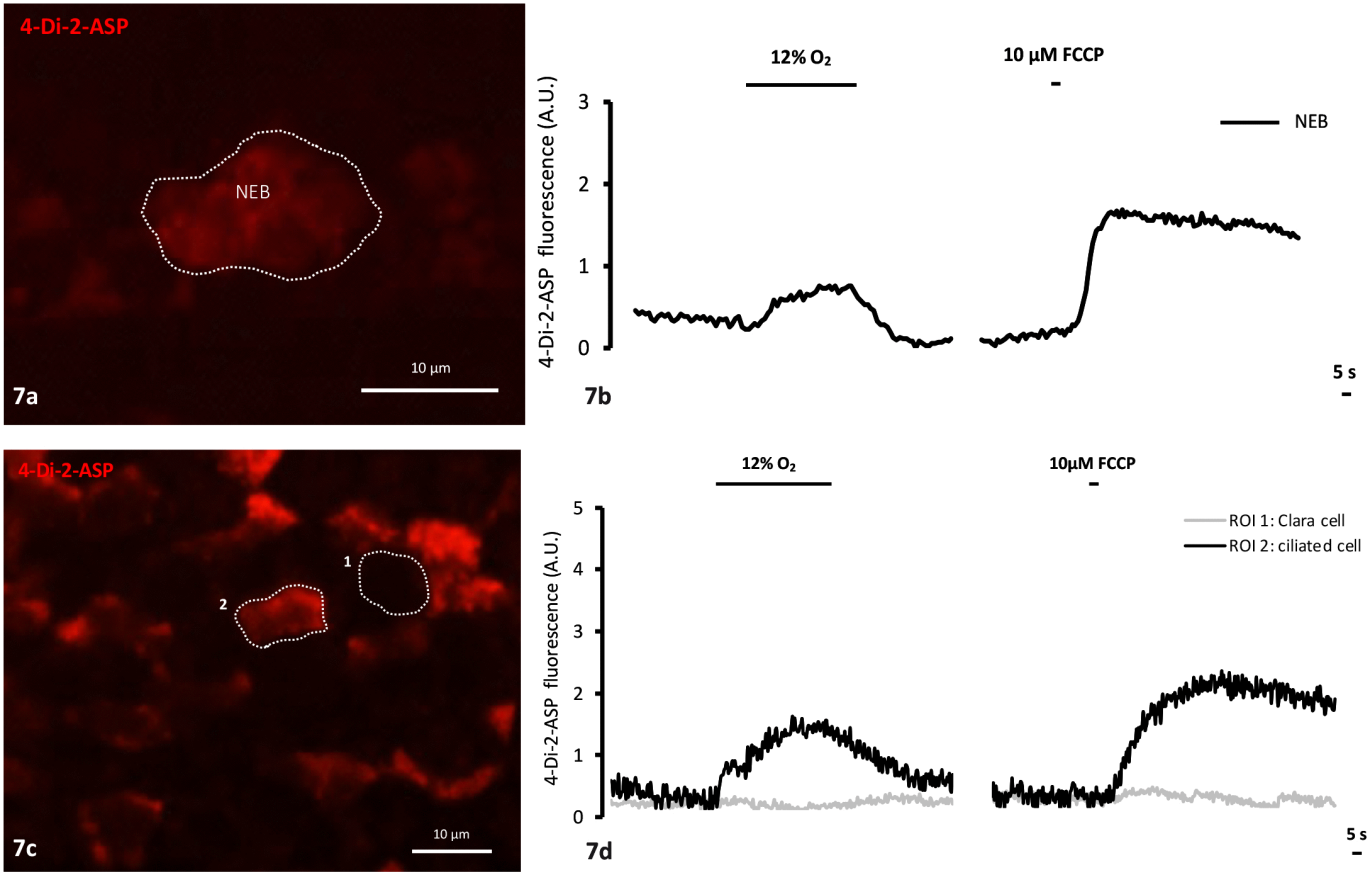
Hypoxic challenge: imaging of mitochondrial membrane potential. Representative recording of changes in 4-Di-2-ASP fluorescence in a NEB (a, b) and airway epithelial cells (c, d) in murine lung slices (wt Bl6; PD20) following perfusion (60 s) with a moderately hypoxic solution (12% O_2_). **a.** 4-Di-2-ASP staining recorded at the beginning of the experiment, showing a NEB (*encircled*) that can be differentiated from the other airway epithelial cells. **b.** Time course of 4-Di-2-ASP fluorescence intensity. NEBs respond to the decline in pO_2_ with a cytoplasmic rise in 4-Di-2-ASP fluorescence, indicating depolarization of the mitochondrial membrane potential. Subsequent control stimulation with the mitochondrial uncoupler FCCP (10µM, 5 s), resulting in a forced depolarization of the mitochondrial membrane, confirms a proper loading of the NEB. **c.** 4-Di-2-ASP staining recorded at the beginning of the experiment, showing non-fluorescent Clara cells (ROI 1) that can be differentiated from the fluorescent ciliated epithelial cells (ROI 2). **d.** Time course of 4-Di-2-ASP fluorescence intensity. Ciliated cells, but not Clara cells, respond to the decline in pO_2_ with a cytoplasmic rise in 4-Di-2-ASP fluorescence, indicating depolarization of the mitochondrial membrane potential. Subsequent stimulation with the mitochondrial uncoupler FCCP (10µM, 5 s), induces comparable 4-Di-2-ASP fluorescence changes.

Acute hypoxic challenges (moderate or severe) elicited an increase in 4-Di-2-ASP fluorescence in prenatal and postnatal NEBs from both wild-type [GD 17−20 (n = 3); PD14−20 (n = 3)] and GAD67-GFP mice (PD14−20; n = 3), indicative of mitochondrial membrane depolarization (Fig 7a,b). After returning to the normoxic state, 4-Di-2-ASP fluorescence returned rapidly to baseline levels. Subsequent application of the mitochondrial uncoupler FCCP (10µM, 5 s) produced comparable responses but with slower recovery (Fig 7a,b).

Similar to what was seen in NEB cells, the hypoxic challenge also resulted in a rise in 4-Di-2-ASP fluorescence in ciliated cells (Figs 7c,d). No changes in fluorescence intensity were observed in Clara cells, which apparently did not take up the 4-Di-2-ASP dye (Figs 7c,d).

### Influence of short-term intermittent hypoxia on the NEB ME

**Fig 8:**
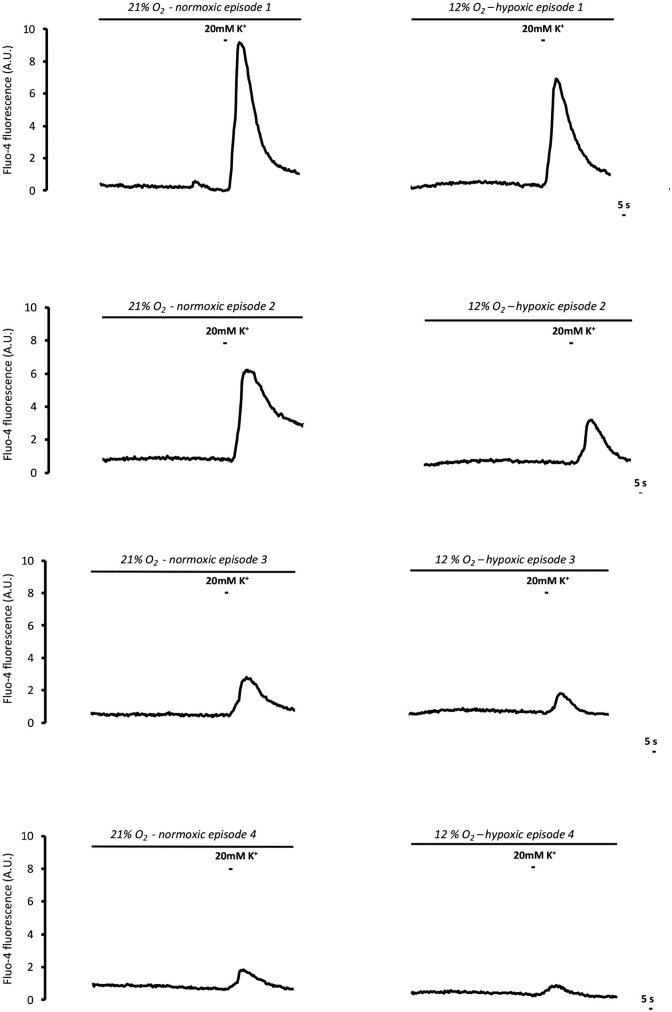
Intermittent hypoxia: calcium imaging and excitability of NEBs. Representative recording of changes in Fluo-4 fluorescence in NEB cells in a 4-Di-2-ASP stained, Fluo-4 loaded murine lung slice (wt Bl6; PD14) during challenge with intermittent hypoxia (5 min normoxia, 5 min hypoxia 12% O_2_; 4 cycles). During each episode, normoxic and hypoxic, NEB cells were activated with a control high [K^+^]_o_ stimulus (20mM; 5 s). None of the NEBs studied respond with a [Ca^2+^]_i_ increase to the repeating hypoxic episodes per se, but the [Ca^2+^]_i_ rise resulting from the high potassium stimulation gradually decreases.

Lung slices of postnatal mice (PD 14−20; n = 3) were exposed for four consecutive episodes of 5 min to mild (12% O_2_) or severe (2% O_2_) hypoxia, each separated by 5 min of normoxia. Intermittent hypoxia failed to induce any increase in Fluo-4 fluorescence, indicating no [Ca^2+^]_i_ elevation in NEB cells. However, short (5 s) depolarizations with high [K^+^]_o_ during each cycle consistently evoked Ca^2+^ responses, though with progressively reduced amplitude (Fig 8).

**Fig 9:**
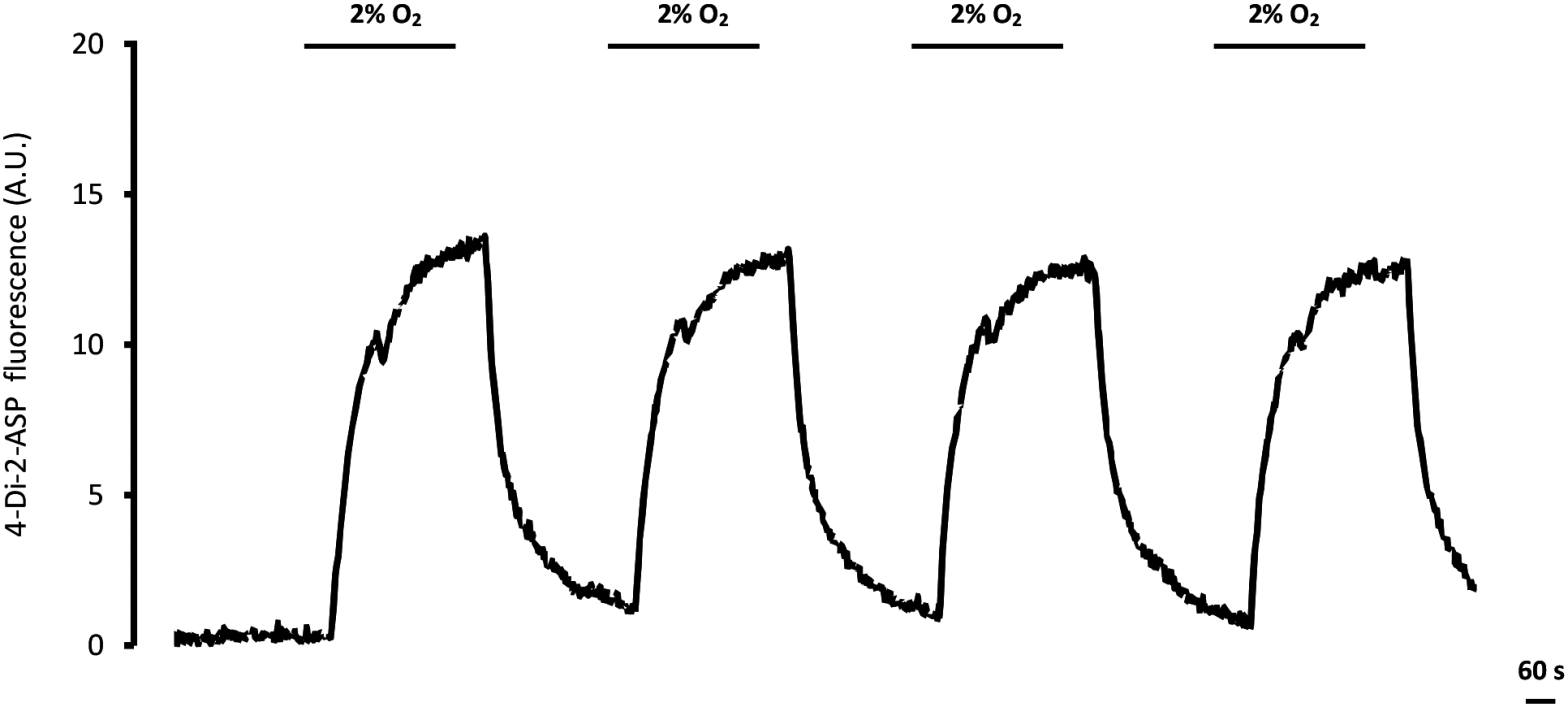
Intermittent hypoxia: imaging of mitochondrial membrane potential in NEBs. Representative recording of changes in 4-Di-2-ASP fluorescence in NEBs during intermittent hypoxia (4 cycles of 5 min) in murine lung slices with severely hypoxic solutions (2% O_2_). NEBs repeatedly respond to a drop in pO_2_ with a cytoplasmic rise in 4-Di-2-ASP fluorescence, indicative of a depolarization of the mitochondrial membrane potential, without apparent potentiation or attenuation of the reaction over time.

Repeated hypoxic episodes produced highly reproducible rises in 4-Di-2-ASP fluorescence in NEBs of postnatal mice (PD14–20; n = 3), signifying mitochondrial membrane depolarization without cumulative potentiation or attenuation across cycles (Fig 9). Similar responses were recorded in ciliated epithelial cells.

### ROS production in mouse NEBs: a role for NADPH oxidase?

The proposed oxygen-sensing mechanism in NEB cells relies on the unavailability of oxygen to be used by NADPH oxidase (NOX2) to generate ROS, thereby creating a drop in ROS production during airway hypoxia.

**Fig 10:**
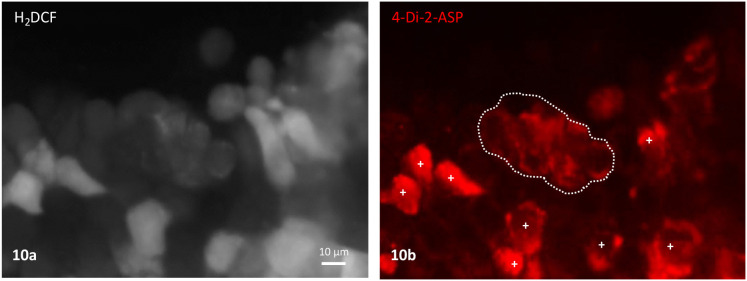
ROS imaging: airway epithelium in lung slices. Incubation of an *ex vivo* lung slice (wt Bl6; PD16) with the ROS indicator H_2_DCF **(a)** and with 4-Di-2-ASP **(b)**. **a.** H_2_DCF reveals polygonal epithelial cells with an intense baseline fluorescence, intermingled with less fluorescent rounded cells. **b.** 4-Di-2-ASP labeling demonstrates a compact group of small 4-Di-2-ASP fluorescent cells, known to represent a pulmonary NEB (*encircled*), typically surrounded by a rim of non-fluorescent CLCs. NEB cells are never contacted by 4-Di-2-ASP fluorescent ciliated cells (*crosses*), which represent an extensive population and largely correspond to the strongly H_2_DCF fluorescent cells.

**Fig 11:**
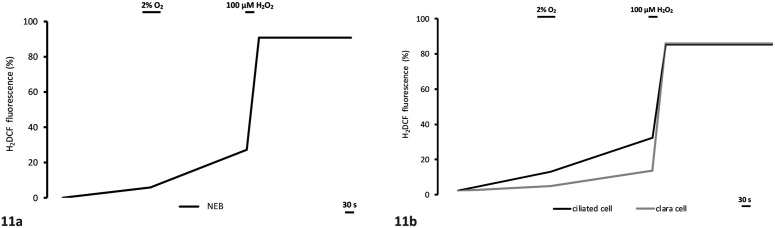
Hypoxic challenge: ROS imaging in NEBs, ciliated cells and Clara cells. Representative recording of changes in H_2_DCF fluorescence measured in a NEB **(a)**, a ciliated cell **(b)** and a Clara cell **(b)** following short-term perfusion of murine lung slices (wt Bl6; PD16) with a severely hypoxic solution (2% O_2_; 60 s) and H_2_O_2_ (100µM; 30s). Both NEBs and other epithelial cells appear to respond to a decrease in O_2_ availability with a small but reproducible increase in H_2_DCF fluorescence intensity, as indicated by a change in the slope of the fluorescence rise after exposure to hypoxia. The positive control challenge with H_2_O_2_ shows that all cells were properly loaded with the ROS probe.

To visualize ROS production in pulmonary NEBs and airway epithelium, live lung slices of postnatal mice (PD14−20; n = 3) loaded with 4-Di-2-ASP, were additionally loaded with the green-fluorescent ROS indicator H_2_DCF (Fig 10a; normoxia). Under normoxic conditions, microscopic investigation revealed a population of polygonal epithelial cells that emitted a much brighter fluorescence than the remainder of the more rounded epithelial cells (Fig 10a). According to the 4-Di-2-ASP staining, the strongly fluorescent cell population could be identified, and is known to correspond to ciliated cells (Fig 10a,b) [77]. Pulmonary NEBs, identified in the 4-Di-2-ASP channel, did not show bright H_2_DCF fluorescence under normoxic conditions (Fig 10a), indicative of a lower ROS production than ciliated cells. Challenge of lung slices with severely (2% O_2_) or moderately (12% O_2_) hypoxic solutions evoked a small but reproducible increase in H_2_DCF fluorescence in NEB cells (Fig 11a), indicative of higher ROS production under hypoxic conditions. Other epithelial cells (Fig 11b) also showed a rise – although to a lesser extent– in H_2_DCF fluorescence, indicated by a change in the slope of the fluorescence rise after exposure to hypoxia. Subsequent application of H_2_O_2_ (100µM, 30 s) to the lung slices resulted in a further strong increase in H_2_DCF fluorescence in both NEB cells and in all other airway epithelial cells (Fig 11a,b).

**Fig 12:**
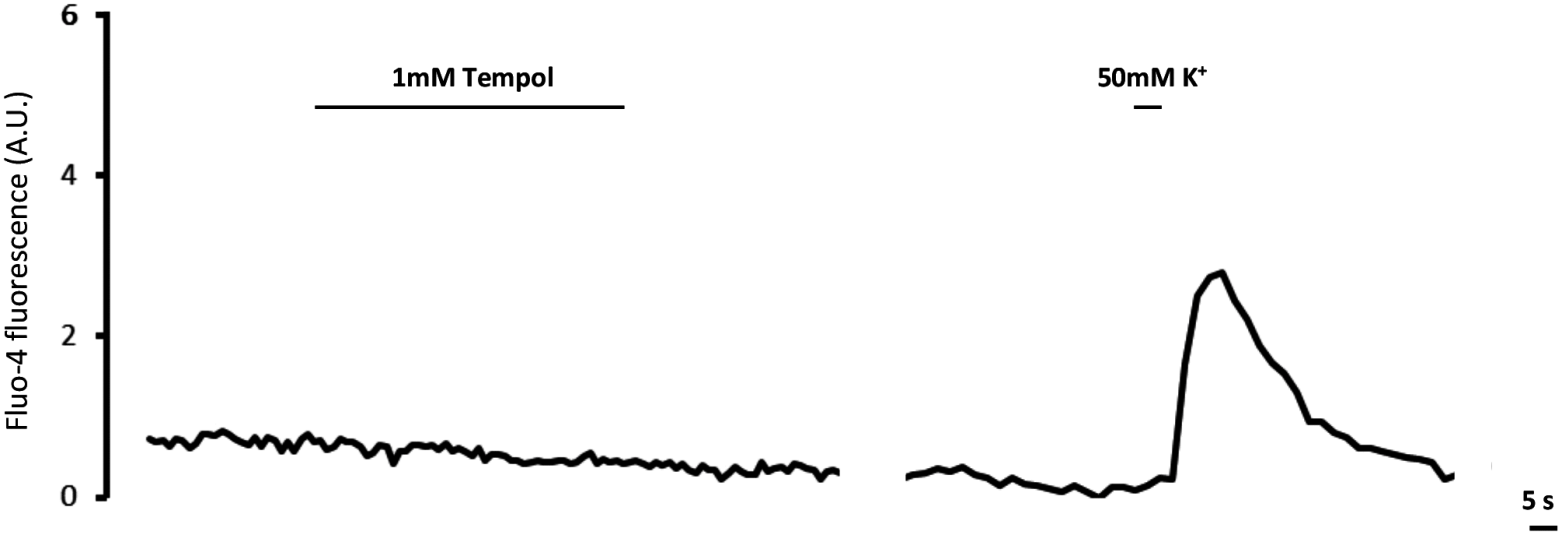
ROS scavenging: calcium imaging in NEBs. Representative recording of changes in Fluo-4 fluorescence in NEB cells in a 4-Di-2-ASP stained, Fluo-4 loaded murine lung slice (wt Bl6; PD14) challenged with 1mM of the membrane permeable ROS scavenger Tempol (60s) under normoxic conditions. NEBs do not respond to application of this ROS scavenger with a rise in [Ca^2+^]_i_, suggesting that the intracellular signaling cascade for neurotransmitter release is not initiated. Subsequent stimulation with high [K^+^]_o_ shows that the studied NEB was viable and properly loaded with the Ca^2+^ indicator.

To mimic the hypothesized hypoxia-induced suppression of ROS production in NEB cells, 4-Di-2-ASP and Fluo-4 loaded lung slices of postnatal mice (PD14−20; n = 3) were exposed to the membrane permeable ROS scavenger Tempol (100 µM - 10 mM). In theory, this superoxide dismutase mimetic would remove the ROS produced by NADPH oxidase in NEB cells, leading to a decrease in ROS concentration, closure of ROS-sensitive K^+^ channels, plasma membrane depolarization, and eventually a rise in [Ca^2+^]_i_. However, none of the tested Tempol concentrations triggered increases in Fluo-4 fluorescence, whereas subsequent depolarization with 50 mM [K^+^]_o_ reliably induced [Ca^2+^]_i_ transients, confirming NEB cell viability (Fig 12).

### Gene expression of NADPH oxidase subunits

**Fig 13:**
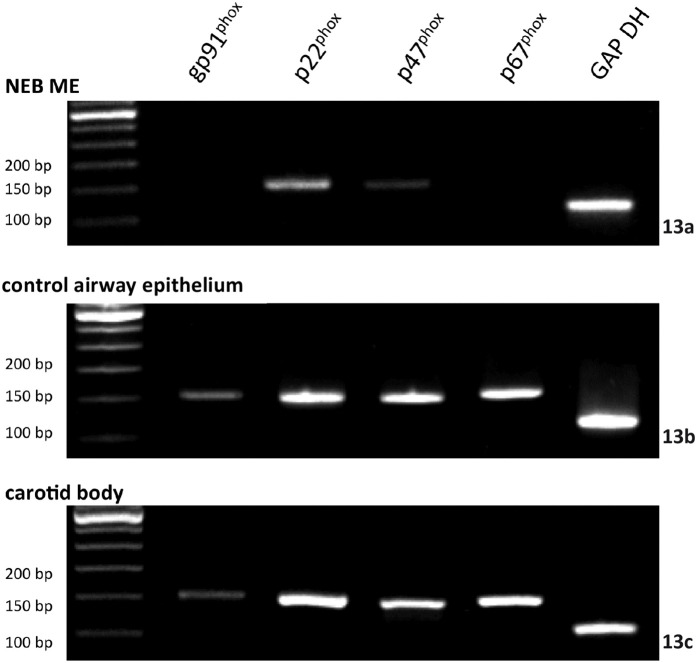
Gene expression of NADPH oxidase subunits. RT-PCR analysis for four components of NADPH oxidase: gp91^phox^ (lane 1), p22^phox^ (lane 2), p47^phox^ (lane 3), p67^phox^ (lane 4) and the housekeeping gene GAPDH (lane 5) in laser microdissected samples of NEB microenvironment (NEB ME) **(a)**, intrapulmonary control airway epithelium (**b**) and carotid body **(c)**. The left lane shows the DNA ladder (bp: base pairs). In the NEB ME, mRNA of only two of the four subunits, p22^phox^ and p47^phox^ (weak), is seen while expression of gp91^phox^ and p67^phox^ is absent. The carotid body and control airway epithelium express all four components.

RT-PCR analysis of LMD samples of the NEB ME from mouse lung cryosections (GAD67-GFP mice; PD16; n = 5) revealed expression of p22^phox^ and p47^phox^ transcripts, but not of gp91^phox^ (NOX2) or p67^phox^ (Fig 13a). In contrast, control airway epithelium expressed all four NADPH oxidase components, including gp91^phox^ (Fig 13b). The mouse carotid body (positive control; wt Bl6; PD16; n = 5) also displayed robust expression of all four subunits (Fig 13c). GAPDH, used as a reference gene, was consistently expressed and no-template controls showed no amplification.

## Discussion

### Lung slice models

*Ex vivo* precision-cut lung slices (PCLSs) are now widely used for various applications and have become standard tools for studying lung physiology in both health and disease [for review see 47,80, 81]. Also functional studies on NEBs have been conducted on PCLCs across multiple species, including mice [40,43–45,48,49,62,77,78,82]. NEBs consist of clusters of pulmonary neuroendocrine cells (PNECs) that are widely dispersed within the airway epithelium, making live lung slices invaluable for studying NEBs in their ‘natural environment’, i.e., selectively innervated and surrounded by specific neighboring cells and tissues.

By combining *ex vivo* lung slices with functional fluorescent indicators in a live cell imaging (LCI) set-up, models for studying NEB function have been optimized in wild-type postnatal mice [40,77,78], in mice with GFP-fluorescent NEBs [43], and in prenatal mouse lungs [45]. These models enable simultaneous, real-time monitoring of physiological changes in multiple NEB cells and their interactions with surrounding cells and tissues. Using this approach, it has been demonstrated that the NEB ME (i.e. PNECs, CLCs and intrinsic nerve terminals) can act as a receptor end-organ, capable of detecting mechanical and chemical changes [for review see 26,48,49]. These stimuli trigger Ca^2+^-mediated ATP release from NEB cells, which can be visualized indirectly through the delayed activation of surrounding CLCs, serving as a functional readout of neurotransmitter release [48,49].

### Carotid body: Proof of concept

In order to confirm that our NEB LCI models could realistically register changes in response to hypoxia, we created an identical confocal LCI set-up to directly visualize cell activation in the mouse carotid body. The carotid body is a well-established chemoreceptor organ that senses changes in O_2_ levels in arterial blood, triggering Ca^2+^-mediated responses, neurotransmitter release, and communication with brain stem neurons that are involved in the regulation of breathing [for review see 83–88]. Our LCI experiments confirmed that depolarizing stimuli led to a rise in a [Ca^2+^]_i_ in glomus cells of the mouse carotid body. Moreover, the increase in [Ca^2+^]_i_ during hypoxic challenges could be clearly visualized, confirming the O_2_-sensing properties of mouse carotid body glomus cells and validating our set-up’s capacity to detect [Ca^2+^]_i_ changes during hypoxic stress. When carotid bodies were loaded with 4-Di-2-ASP, the red-fluorescent dye used to label NEBs in wt Bl6 mice, glomus cell clusters still showed [Ca^2+^]_i_ increases during hypoxia, indicating that 4-Di-2-ASP does not interfere with the O_2_-sensing mechanism in mouse carotid body cells.

### Hypoxia and calcium imaging in the NEB microenvironment

In none of our presented murine lung slice models, hypoxia resulted in a [Ca^2+^]_i_ rise in NEB cells, while subsequent positive control stimuli confirmed that the NEBs were viable, properly loaded, and capable of releasing neurotransmitters. NEBs have been proposed to function as auxiliary chemoreceptors for the not yet fully developed carotid bodies during late fetal and perinatal stages, and might lose this capacity postnatally [37]. In our prenatal mouse lung slice model, acute hypoxia also failed to induce Ca^2+^-mediated activation and neurotransmitter release of fetal NEB cells. Since GAD67-GFP mouse lung slices inherently express GFP in NEB cells [43], and do not require initial staining with 4-Di-2-ASP [78], the observation that acute hypoxia did not trigger a rise in [Ca^2+^]_i_ indicates that 4-Di-2-ASP was not responsible for the lack of hypoxic activation in NEB cells. Therefore, it appears that acute hypoxia is unable to induce a [Ca^2+^]_i_ rise in mouse NEB cells. Although previous studies have shown that hypoxia attenuates outward K^+^ currents in NEB cells and related cell models [55–57,61,62], our findings strongly suggests that such changes may be insufficient to cause membrane depolarization, voltage-gated Ca^2+^ entry, and subsequent neurotransmitter release from NEB cells in their natural microenvironment. These observations contrast with functional studies in neonatal rabbits [33] and postnatal hamster lung slices [89], in which quantal release of serotonin (5-HT) was measured using carbon fiber amperometry, and exocytosis appeared to depend on extracellular Ca^2+^ entry.

Control stimuli applied during recurrent hypoxia revealed a decline in NEB cell excitability, a pattern inconsistent with the proposed hypoxia-sensing mechanism (for review see [13]), which suggests that hypoxia should depolarize and activate the PNECs. On the other hand, low O_2_ levels did lead to mitochondrial membrane depolarization and increased ROS levels, indicating that NEB cells are affected by hypoxia. However, these responses were also observed in other airway epithelial cells, suggesting a non-selective mechanism in response to hypoxia that is in no way associated with exocytosis.

Prolonged exposure to intermittent hypoxia has been shown to enhance (long-term facilitation) the response of carotid body glomus cells to subsequent acute hypoxia in adult rats [90] and mice [91]. Since NEB cells in our models did not show measurable Ca^2+^-mediated responses to acute hypoxia, lung slices were also exposed to intermittent hypoxia in an attempt to potentiate the NEB response. Intermittent hypoxia induced reproducible and reversible mitochondrial membrane potential depolarization in NEB cells during each episode, but no detectable increase in [Ca^2+^]_i_, further arguing against activation of NEB cells and Ca^2+^-mediated exocytotic neurotransmitter release. Additionally, NEB cell excitability during normoxic episodes gradually declined over time during intermittent hypoxia, supporting the idea that intermittent hypoxia is more challenging for NEB cells than acute hypoxia.

### The oxygen sensor: NADPH oxidase and ROS?

The first step of the originally suggested hypoxia-signaling mechanism in PNECs involves membrane-bound NADPH oxidase and the reduced availability of O_2_ substrates, which inhibits ROS production [50]. If this were the case, scavenging normoxically produced ROS with antioxidants in lung slices should mimic hypoxia. However, Tempol, a potent membrane permeable ROS scavenger, did not induce a rise in [Ca^2+^]_i_ in NEB cells in our study. Furthermore, the use of the fluorescent ROS-indicator H_2_DCF in mouse lung slices, showed a small but reproducible increase in ROS levels upon acute hypoxic challenge, but this was also observed in other airway epithelial cells.

RT-PCR on pooled LMD samples of the NEB ME from mouse lung cryosections revealed expression of two NADPH oxidase subunits, p22^phox^ and p47^phox^ (weak), in the NEB ME, but no mRNA for gp91^phox^ or p67^phox^. In contrast, LMD samples of CAE and carotid body glomus cells express all four verified NADPH oxidase components, including gp91^phox^, which has been reported in NEB cells of fetal rabbits and human infants and in small cell lung carcinoma cell lines (see [92]). Our results align with recent data using single cell RNA sequencing of mouse PNECs, which did not show PNEC expression of NADPH oxidase subunits p91phox, p47phox or p67phox, while p22phox was broadly expressed in all cells [39]. In our study, mouse carotid bodies clearly express all four components of NADPH oxidase. Because gp91^phox^ is a necessary unit to compose a functional NADPH oxidase [for review see 93], its absence suggests that the NADPH oxidase enzyme complex may not function as an oxygen sensor in the mouse NEB ME.

Potential technical issues or species differences cannot be excluded. However, the most important finding from our RT-PCR experiments is not necessarily the absence (or very low) expression of some of the subunits in NEB cells, but the presence of all NADPH oxidase subunits in control airway epithelium. This finding at least indicates that the suggested NADPH oxidase step in the oxygen sensing pathway is not selective for NEB cells in the airway epithelium.

Recent single-cell RNA data on mouse lungs revealed that K_v_ channels, which have been implicated in the hypoxia-sensing mechanisms in glomus cells, are absent in PNECs [39]. Kuo and coworkers did also not find selective expression of genes associated with the mitochondrial oxygen-sensing pathway in PNECs, concluding that the identity of the acute oxygen sensor in PNECs remains uncertain [39].

Due to their structural similarities, pulmonary NEBs have long been compared to carotid bodies, with glomus cells often cited as a model for oxygen sensing in PNECs. However, current insights challenge the notion that O₂ sensing is mediated solely by specialized K⁺ channels. The identification of multiple types of O₂-regulated K⁺ channels in glomus cells — including voltage-gated, Ca² ⁺ -activated, and background K⁺ channels — makes it unlikely that oxygen sensing depends exclusively on direct or indirect modulation of a specific K⁺ channel [for review see 83]. Moreover, alternative O₂-sensing mechanisms in glomus cells —such as AMP kinase activation, production of gasotransmitters (e.g., carbon monoxide and hydrogen sulfide), and the activation of a lactate-sensitive olfactory receptor (Olfr78)— have shifted focus away from NADPH oxidase as the primary O₂ sensor. Importantly, none of these proposed pathways are essential for acute O₂ sensing, as mice lacking the relevant genes still show normal carotid body responses to hypoxia [for review see 83]. Given these unresolved questions about oxygen sensing in carotid bodies, it is reasonable to propose that oxygen-sensing mechanisms in NEBs may be similarly complex and less linear than initially assumed.

### Hypoxia-induced secretion by NEBs?

The proposed mechanism for hypoxia signaling to the CNS via pulmonary NEBs is primarily based on the release of serotonin (5-HT). A variety of techniques and model systems (*in vivo*, *ex vivo*, *in vitro)* have been employed to expose animals, lung slices, NEBs, isolated NEB cells or SCLC-derived cell lines to hypoxia. These studies consistently demonstrated increased exocytosis of basally located dense-cored vesicles (DCVs) in NEB cells (based on electron microscopic interpretation), accompanied by a decrease in intracellular 5-HT content (assessed by formaldehyde-induced fluorescence in lung sections of neonatal rabbits exposed to hypoxia [94], and by HPLC analysis of isolated cultured fetal rabbit NEB cells [95]). Corresponding increases in extracellular 5-HT levels were detected using ELISA in tumor cell models [96] and carbon fiber amperometry in newborn rabbit [33] and hamster lung slices [89]). Under normoxic conditions, exposure to a Ca^2+^ ionophore —which increases intracellular Ca^2+^— also caused a reduction in intracellular 5-HT and promoted exocytosis of DCVs in NEB cells. These findings indicated that stimulus-secretion coupling is Ca^2+^-dependent [95]. Collectively, these observations suggest that hypoxia enhances basal exocytotic 5-HT release in NEB cells of fetal and neonatal rabbit lungs, as well as in certain tumor cell models.

However, the present study’s finding that hypoxia does not induce Ca² ⁺ -mediated exocytosis in mouse NEB cells supports the hypothesis that alternative, non-Ca² ⁺ -dependent transmitter release mechanisms may contribute to how PNECs transduce hypoxic stimuli.

In the current study, repeated hypoxic exposure accompanied by control stimuli revealed a progressive weakening of NEB cell excitability. This observation aligns with several reports describing hypoxia-induced inhibition of NEB cell secretory activity [97–100]. Additional indirect evidence stems from studies showing that acute, chronic or intermittent hypoxia increases CGRP concentrations in NEB cells [100–102], without corresponding changes in CGRP mRNA levels or NEB cell numbers [102,103], suggesting suppression of CGRP-release. In a pilot study, we attempted to directly quantify differences in CGRP release from NEB cells by performing CGRP-ELISA on collected physiological solution of murine PCLSs exposed to either normoxic or hypoxic conditions. Since the data were inconclusive, mainly due to the detection limit of the assays, the proposed paracrine mechanism still requires future validation. Given that NEBs contain both 5-HT (a potent pulmonary vasoconstrictor) and CGRP (a potent vasodilator), their role during hypoxia may primarily involve the local regulation of pulmonary blood flow via reciprocal release of these bioactive peptides, rather than direct signaling to the CNS [25,53].

### *In vivo* pulmonary effects of hypoxia

While our results may appear to contradict the prevailing literature describing pulmonary NEBs as hypoxia sensors and rapid signalers to the brain [for reviews see 25,37], they are consistent with findings from electrophysiological airway sensory receptor studies [for review see 7,18,104]. Many single-fiber recording studies from airway-related vagal afferent nerves over the years, have generally shown that activity in these fibers is unaffected by airway hypoxia. Recent investigations [39,105–107] and reviews [53] have similarly proposed that PNECs – long thought to serve both as local signaling centers and as rapid conductors of sensory information to the brain – may in fact be limited to a local role in lung physiology with respect to hypoxia. Emerging research using cutting-edge technologies has highlighted additional functions of PNECs, including their involvement in amplifying asthmatic responses, promoting tissue regeneration, and serving as potential cells of origin for certain lung cancers [53]. Responses to sustained and/or chronic hypoxia appear to involve prolyl hydroxylase (PHD)- and hypoxia-inducible factor (HIF)-dependent mechanisms, leading to NEB hyperplasia in the lungs of Phd^-/-^ mice [108].

## Conclusion

Using the mouse carotid body as a proof of concept, we confirmed that our confocal LCI set-up can visualize hypoxia-induced [Ca^2+^]_i_ changes in oxygen-sensing cells. However, in none of the applied LCI models, hypoxia induced a Ca^2+^-mediated response of NEB cells or subsequent exocytotic neurotransmitter release. This is further supported by the non-selective and seemingly non-functional expression of NADPH oxidase components in mouse NEBs. On the other hand, hypoxia did compromise NEB cell excitability, suggesting a potential local role for NEBs related to airway oxygenation. At present, a potential bias of developmental or species-specific differences cannot be excluded.

Altogether, the presented results strongly argue for reconsideration of the proposed straightforward hypoxic vagal signal transduction pathway from pulmonary NEBs to the CNS and support the idea that rather local paracrine actions may be essential for balancing homeostasis to airway oxygenation.

## Supporting information

S1 Fig Gene expression of NADPH oxidase subunits. Original uncropped and unadjusted images of the gel blot.
(PDF)
