# Supplementary figures and images for: Are pulmonary neuroepithelial bodies sensors for acute airway hypoxia implicated in the central regulation of breathing?
Source: PLoS One. 2026 Jun 23;21(6):e0351688. doi: 10.1371/journal.pone.0351688 (PMC13289943; doi:10.1371/journal.pone.0351688)

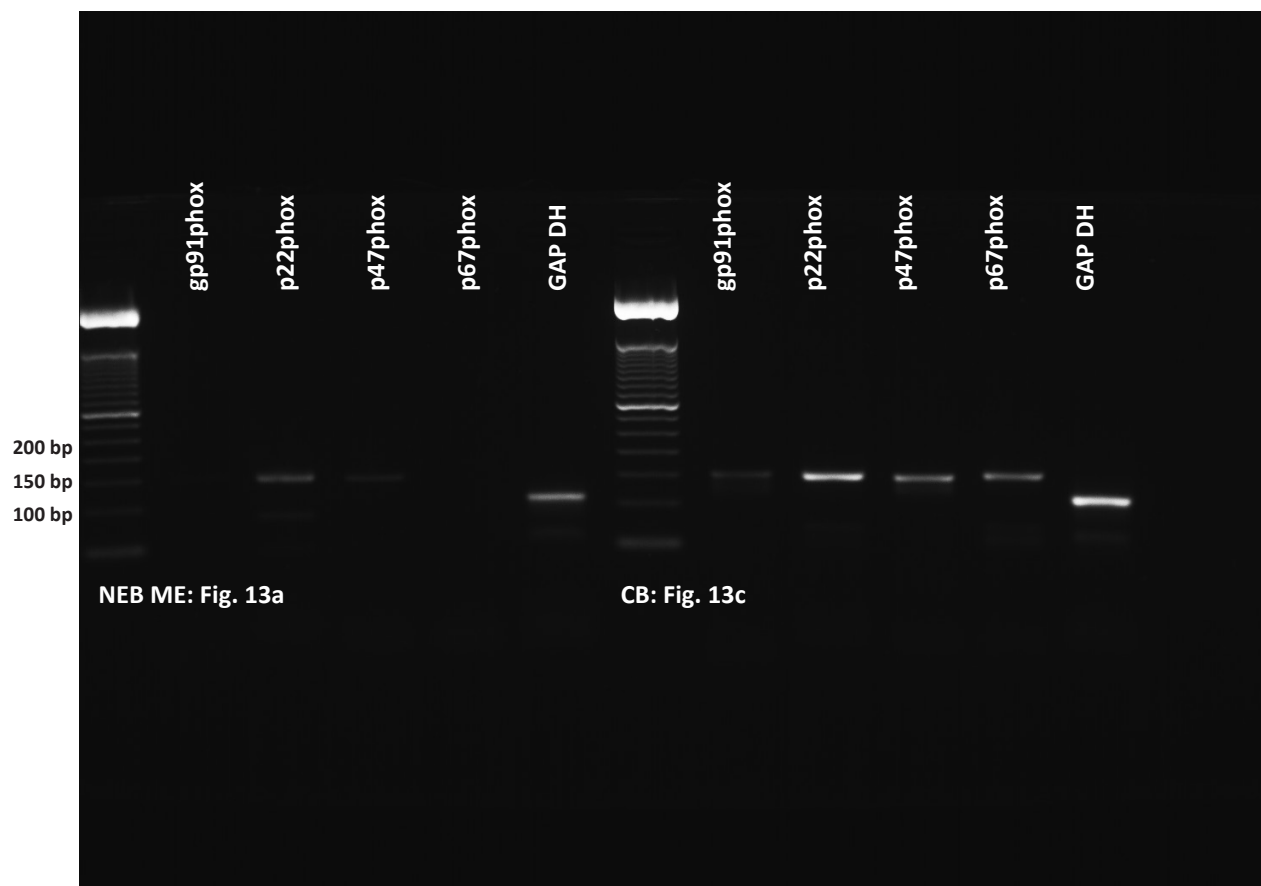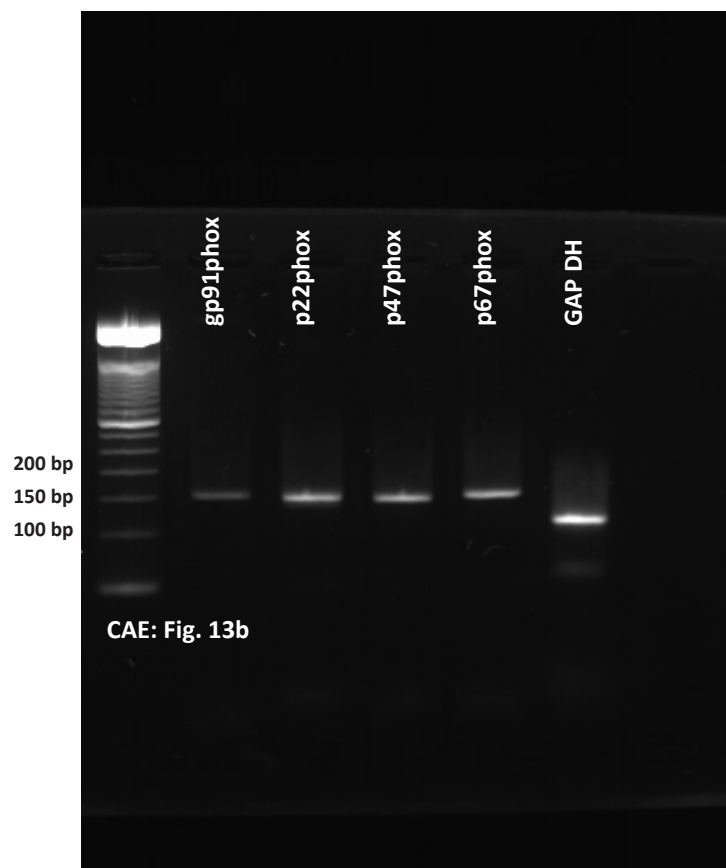

Supplement: S1 Fig — Original uncropped and unadjusted images of the gel blot. (PDF) [file pone.0351688.s001.pdf]
